# Confirmation of a Useful Dark‐Room Resting‐State Procedure: Periodic and Aperiodic MEG Activity in Children

**DOI:** 10.1111/psyp.70261

**Published:** 2026-02-22

**Authors:** Marybeth McNamee, Heather L. Green, Guannan Shen, Marissa DiPiero, Drayton L. Murray, Mia Pearce, Alice Onyango‐Opiyo, Song Liu, Lisa Blaskey, Emily S. Kuschner, Mina Kim, Rose E. Franzen, Gregory A. Miller, Yuhan Chen, J. Christopher Edgar

**Affiliations:** ^1^ Lurie Family Foundations MEG Imaging Center, Department of Radiology The Children's Hospital of Philadelphia Philadelphia Pennsylvania USA; ^2^ Department of Radiology, Perelman School of Medicine University of Pennsylvania Philadelphia Pennsylvania USA; ^3^ Center for Autism Research, Department of Pediatrics The Children's Hospital of Philadelphia Philadelphia Pennsylvania USA; ^4^ Department of Psychiatry, Perelman School of Medicine University of Pennsylvania Philadelphia Pennsylvania USA; ^5^ Department of Psychology University of California Los Angeles California USA; ^6^ Department of Psychiatry and Biobehavioral Sciences University of California Los Angeles California USA; ^7^ Department of Psychology and Beckman Institute University of Illinois Urbana‐Champaign Champaign Illinois USA

**Keywords:** alpha power, aperiodic, autism spectrum disorder, magnetoencephalography, peak alpha frequency, periodic, resting‐state alpha, typically developing

## Abstract

In a previous paper, we showed in children 6–12 years old that a resting‐state (RS) eyes‐open dark room (DR) task provides RS parietal‐occipital alpha measures similar to those obtained using the standard RS eyes‐closed (EC) exam. Results provided initial evidence that the RS DR procedure is feasible and useful with populations often excluded from electrophysiology RS studies, such as participants who cannot remain awake with their eyes closed or cannot remain still for an extended period. The present study extended the DR and EC comparisons to a much larger sample of children spanning a wider age range and expanded the analysis strategy to examine RS aperiodic measures (offset and slope [exponent] of the power spectrum) and to evaluate 15 distinct brain regions rather than just the previously examined parieto‐occipital RS periodic alpha activity. RS activity was recorded using MEG, here reporting on 147 DR and EC datasets obtained from children (including 23 with evaluable datasets at multiple timepoints) with typical development (TD; *N* = 69) and children with autism spectrum disorder (ASD; *N* = 53) 7.7–17.1 years old. Findings showed good reliability in both TD and ASD for the EC and DR parietal‐occipital peak alpha frequency (frequency with highest alpha power; interclass correlation [ICC] = 0.84, *p* < 0.001). The ICC for periodic parieto‐occipital PAF power was lower (ICC = 0.65). For offset and exponent, the two RS aperiodic measures, fair to good reliability for both groups was observed between DR and EC at all 15 brain regions (mean and median ICC values 0.77–0.80). Offset and exponent values differed significantly across the 15 brain regions, as did associations between age and both aperiodic measures. Findings confirm that the DR exam is a viable way to obtain RS periodic and aperiodic measures. The lack of TD/ASD differences in the EC and DR periodic and aperiodic ICCs supports the generalizability of the DR procedure. Finally, regional differences in aperiodic measures demonstrate the need to assess aperiodic activity in brain source space rather than scalp sensor space.

## Abstract 2

Resting‐state (RS) brain activity, when an individual is not pursuing a specific goal, provides a foundation for basic and higher‐order brain processes relied on when people are focused on a goal. RS activity is typically examined in an eyes‐closed (EC) condition. Unfortunately, the RS EC exam can only be used with individuals who can follow instructions to stay awake with their eyes closed for an extended period. That excludes groups such as infants, toddlers, and individuals who have intellectual disability. Comparison of RS neural measures obtained in an EC condition and in a dark‐room (DR) eyes‐open condition in children with typical development and children with autism spectrum disorder (7.1–17.1 years old) showed that the DR eyes‐open exam is a viable way to obtain RS measures, with some caveats about how the brain activity should be measured.

## 1 Introduction

Resting‐state (RS) functional brain measures such as alpha‐band power and peak alpha frequency (PAF; in adults the frequency from 8 to 12 Hz showing the highest alpha activity) provide information regarding the maturation of neural circuits (Rempe et al. 2023; Klimesch 1999; Candelaria‐Cook et al. 2022; Cragg et al. 2011). RS alpha measures have also informed our understanding of neural‐circuit activity in clinical conditions such as schizophrenia (Canive et al. 1998, 1996; Miyauchi et al. 1996, 1990; Itil et al. 1975; Small et al. 1984), autism spectrum disorder (Cornew et al. 2012; Edgar et al. 2015; Cantor et al. 1986; Dawson et al. 2007), dementia (Lopez‐Sanz et al. 2016),^1^ and epilepsy (Gundel 1983). As RS alpha activity is highest when an individual is at rest with their eyes closed, electrophysiology studies typically obtain RS measures with the eyes closed (Klimesch 1999; Berger 1929; Adrian and Matthews 1934; Edgar et al. 2023). A limitation of the RS eyes‐closed (EC) exam is that it requires that the participant remain awake with their eyes closed for several minutes. Given that individuals with intellectual disability can have difficulty complying with task instructions, these individuals are often excluded from RS studies. Given that infants are unable to keep their eyes closed for an extended period of time without falling asleep, infant RS measures are typically obtained while the infant/toddler is at rest with the eyes open, sometimes viewing visual stimuli (Carter Leno et al. 2021; Schaworonkow and Voytek 2021; Karalunas et al. 2022; Favaro et al. 2023; Vandewouw et al. 2024; Wilkinson et al. 2024; Stroganova et al. 1999).

When studying RS alpha‐band activity, the use of an eyes‐open RS task is limited, as there is little or no RS alpha activity when the eyes are open (Schaworonkow and Voytek 2021; Cellier et al. 2021; Hill et al. 2022; Isler et al. 2023). Research from the 1940s to the late 1990s suggests an alternative method for obtaining RS EC alpha measures (Berger 1929; Adrian and Matthews 1934; Mulholland 1968, 1995; Chapman et al. 1970). In particular, researchers have noted that the RS alpha rhythm is present when a participant is in a completely dark room with their eyes open. Berger first reported this phenomenon in 1929 (Berger 1929), with Adrian and Matthews a few years later noting that, “After some minutes in the dark the [alpha] rhythm is present with the eyes open. Closing them does not alter the rhythm.” (Adrian and Matthews 1934). Researchers have since reported a similarity between RS eyes‐closed and dark‐room (DR) eyes‐open alpha activity (Mulholland 1968, 1995; Ellingson 1956). For example, Chapman et al. (1970) reported that posterior alpha activity is present when the eyes are open in a completely dark room and that this activity is greatly diminished when the eyes are open and the lights are on.

In Edgar et al. (2023) we showed good reliability between the RS DR and EC alpha measures in children with and without autism spectrum disorder (ASD). In particular, findings showed good reliability for the RS DR and EC PAF (interclass correlation [ICC] = 0.83), with lower ICCs observed for PAF power (ICCs in the 0.70s). No differences in the DR and EC ICCs were observed between typically developing (TD) and ASD groups. Finally, age was associated with DR and EC PAF in both groups. The present study builds on Edgar et al. (2023), here reporting on the RS DR and EC alpha measures in an overlapping but larger sample of children with and without autism and spanning a larger age range. Given that RS neural activity consists of periodic activity (e.g., PAF) as well as RS aperiodic activity (e.g., quantified as offset and exponent, described below), DR and EC aperiodic measures were also compared. For all analyses, RS periodic and aperiodic measures were examined in brain source space. In the present study, support for the use of the DR task would be demonstrated via showing (1) high ICCs and no statistical difference in the ICCs between groups, and (2) demonstrating the same group differences in periodic (and perhaps aperiodic activity) in the EC and DR conditions. The text below briefly reviews the RS periodic and aperiodic measures employed in the present study.

### 1.1 RS Periodic Alpha‐Band Activity

In adults in a relaxed and awake state with their eyes closed, 8–12 Hz alpha oscillations are the dominant rhythm, most prominent in parietal‐occipital regions (Edgar et al. 2015; Berger 1929; Huang et al. 2014; Haegens et al. 2014; Salmelin and Hari 1994). Researchers are interested in RS alpha activity as it: (a) indicates a brain at rest but ready to be engaged (Klimesch 1999; Adrian 1943), (b) is associated with primary sensory activity (Neuper et al. 2006; Weisz et al. 2011), (c) is associated with working memory and processing speed (Klimesch 1999; Edgar et al. 2019; Klimesch et al. 1996), (d) is thought to be central to how local networks process information, likely via the coupling of activity across frequencies (Osipova et al. 2008; Sauseng et al. 2008), and (e) is thought to be essential to top‐down cognitive control processes (Hwang et al. 2014; van Diepen et al. 2015).

### 1.2 RS Aperiodic Activity

Recently, scientists have called into question previous EEG and MEG RS findings, noting that most RS power‐spectrum analyses conflate two brain processes, because RS neural activity exhibits aperiodic as well as periodic activity. Typical power analysis models a time series as a sum of sinusoids, which is appropriate for modeling periodic activity. To the extent that the neural activity is not entirely periodic, its aperiodic components will be mismodeled as sinusoids, with the consequence of exaggerating the power across all frequencies. Thus, what appear to be peaks in the power spectrum, such as RS alpha activity, may overestimate that periodic activity (He 2014; Freeman and Zhai 2009; Donoghue, Haller, et al. 2020). It has thus been argued that much of the power that appears at lower frequencies in conventional power‐spectrum analyses is actually exaggerated due to mismodeled aperiodic activity. In particular, RS electrophysiology power spectra show a characteristic 1/f power distribution, with power higher for lower frequencies, due to potentially substantial contributions from aperiodic activity. The 1/f phenomenon is often referred to as non‐sinusoidal “background noise” (Bedard et al. 2006; Usher et al. 1995) but is very likely neurally meaningful (Donoghue, Haller, et al. 2020; Donoghue, Dominguez, and Voytek 2020; Ostlund et al. 2022). Thus, it has been argued that RS analyses should distinguish the 1/f aperiodic activity from periodic oscillatory activity (including the PAF) to understand RS neural activity (e.g., see Donoghue, Haller, et al. 2020; Bullock et al. 2003).

Recently developed algorithms provide distinct estimates of RS periodic and aperiodic activity (e.g., the “specparam” toolbox, originally called the FOOOF toolbox [Donoghue, Haller, et al. 2020; Ostlund et al. 2022]). The specparam model provides estimates of: (1) periodic measures including spectral peaks, with center frequency and power computed for each identified peak, and (2) aperiodic measures including the exponent (log of the slope of the 1/f function) and the offset (vertical displacement of the 1/f function). A growing literature demonstrates the need to parameterize the RS power spectrum as specparam does, in order to accurately characterize and distinguish periodic and aperiodic RS maturational changes (Donoghue, Dominguez, and Voytek 2020; Ostlund et al. 2022; He et al. 2019). Studies examining aperiodic activity in children have shown that from childhood to late adolescence the RS 1/f power‐spectrum slope flattens as a function of age until eventually stabilizing at an adult level (Schaworonkow and Voytek 2021; Karalunas et al. 2022; He et al. 2019; Tran et al. 2020; Waschke et al. 2017; Voytek et al. 2015).

Unfortunately, most parameterization efforts to assess RS activity have been problematic because almost all such studies have applied specparam (or similar parameterization methods) to EEG or MEG sensor data, thus assessing power spectra measures that reflect activity from multiple brain regions (for discussions of the problems associated with this approach, see Hoechstetter et al. 2004; Scherg and Berg 1996; Scherg and Picton 1991; Edgar et al. 2017; Edgar et al. 2003). For example, in most pediatric studies aperiodic measures have been obtained from power analyses of an average of EEG sensors (Karalunas et al. 2022; Trondle et al. 2022) or via two or more regional clusters of EEG sensors (Carter Leno et al. 2021; Schaworonkow and Voytek 2021; Favaro et al. 2023; Cellier et al. 2021; Hill et al. 2022; Trondle et al. 2022; Rico‐Picó et al. 2023). Of the studies computing aperiodic measures from regional clusters of scalp sites, Rico‐Picó et al. (2023) and Schaworonkow and Voytek (2021) observed significant regional differences in the aperiodic measures, whereas the remaining studies observed no significant regional differences (Favaro et al. 2023; Cellier et al. 2021; Hill et al. 2022; McSweeney et al. 2023) or did not statistically assess regional differences (Carter Leno et al. 2021). Differences between the above EEG studies that may account for differences in findings are many, including but not limited to the age of the children and EEG sensors examined.

As described above, the specparam algorithm computes two parameters, the exponent and the offset, as a means of quantifying the contribution of aperiodic activity to a power spectrum. To our knowledge, only two MEG studies have explored regional differences in aperiodic exponent and offset activity in source space. He et al. (2019) obtained source‐space measures but then computed a single aperiodic measure, averaging across all sources. Vandewouw et al. (2024) assessed regional differences in age and aperiodic exponent and offset associations in children and adults 1–38 years old. They found that the aperiodic exponent decreased with age across brain regions, with the largest effects in left pre‐central, left post‐central, left superior parietal, left inferior parietal, left and right cuneus, and left and right precuneus.

Building upon Vandewouw et al. (2024) and our own previous study of the dark room method (Edgar et al. 2023), the present study systematically addresses the possibility of regional differences in RS aperiodic activity via examining RS measures in source space derived from MEG. MEG non‐invasively measures electromagnetic neural activity, with the temporal resolution of MEG limited only by the data acquisition rate, thus allowing real‐time (submillisecond) assessment of brain neural activity. MEG, in combination with structural (sMRI), provides good spatial resolution of brain activity via source localization, especially for cortical activity (Miller et al. 2007). Compared to EEG, MEG is less sensitive to conductivity differences between the brain, cerebral spinal fluid, skull, and scalp, and thus MEG is often preferred for source localization (Hamalainen et al. 1993; Chen et al. 2019). For recent discussions on the use of MEG and source localization to study RS activity in children, see Edgar et al. (2023), Rempe et al. (2023), and Ott et al. (2021).

In the present study, DR and EC periodic alpha activity and aperiodic offset and exponent measures were compared, with samples of 69 TD and 53 ASD children versus the 37 TD and 30 ASD children reported in Edgar et al. (2023), and now reporting on a wider age range. Given age‐related PAF changes in children (e.g., see Miskovic et al. 2015; Somsen et al. 1997) as well as age‐related aperiodic changes (Schaworonkow and Voytek 2021; Cellier et al. 2021; Hill et al. 2022; McSweeney et al. 2023; Wilkinson et al. 2023), analyses also assessed the similarity of the associations between age and the EC and DR periodic and aperiodic measures. Given significant overlap in children here and in Edgar et al. (2023), good reliability of the RS periodic measures was expected. Examining the reliability of aperiodic activity in children with and without autism across time (eyes‐open RS exams a median of 6 days apart), Levin et al. (2020) reported poor reliability (ICCs 0.28–0.70; see their table 4). Better reliability was hypothesized in the present study, as the RS data were collected during the same session. Finally, as in Edgar et al. (2023), although a comparison of TD and ASD alpha measures was not the primary focus of this study, group comparisons were conducted to assess the similarity of the DR and EC group findings. It was hypothesized that the pattern of group differences would be the same for the DR and EC data, this again demonstrating the similarity of these two RS tasks.

## 2 Method

This study was approved by the local Institutional Review Board, and all families gave written informed consent. When competent to do so, children 7 years of age and older gave verbal and written assent to participate. Families were compensated for their participation in the study.

### 2.1 Participants

DR and EC data were obtained from children participating in a longitudinal study examining the maturation of brain function and structure (R01MH107506). Children were enrolled in the study between 6.9 and 15.9 years old, with longitudinal brain imaging measures obtained ~1.5, ~3.0, and ~4.5 years after the initial exam (via a continuation of this R01, several new participants were enrolled in this study when they were 10+ years old, and with no brain measures at an earlier age). Whereas all children completed a RS EC exam, the DR exam was not initially part of the study. DR data were first collected ~2.5 years after the start of the study. In addition, given the priority of the RS EC exam, EC data were collected first, with the DR exam administered when time permitted.

Four TD and one ASD DR dataset could not be analyzed, as the trigger information was not collected. As detailed below, 4 children were removed as multivariate outliers. After excluding the above, 147 DR and EC datasets were available from 122 children (69 TD and 53 ASD) 7.7–17.1 years old. Within the sample, 23 children (13 TD, 10 ASD) had evaluable datasets at multiple timepoints, and since the visits occurred more than 1 year apart, all available datasets were retained for analysis. Findings are reported from children with DR + EC data at any time point in the longitudinal study design, specifically including 20 children with DR and EC measures at two timepoints (11 TD, 9 ASD), and 3 children with both measures at three timepoints (2 TD, 1 ASD). Analyses excluding the second or third recordings from these 23 children showed similar patterns of findings as those reported in this manuscript (see Supporting Information).

The TD and ASD children met the following exclusion/inclusion criteria: (a) English as primary language, (b) no history of traumatic brain injury or other significant medical or neurological abnormality, (c) no premature birth (< 34 weeks gestation), (d) no uncorrectable sensory impairments (somatosensory, hearing, visual), (e) no sMRI contraindications, (f) no genetic conditions with a very high incidence of ASD (e.g., 22q/Fragile X), and (g) no neuroleptic/antipsychotic medication. At the time of the MEG exam, eight of the children with ASD were prescribed stimulant medications; stimulants were withheld for at least 24 h, but no more than 36 h, prior to the MEG exams. Two of the children with ASD were taking antidepressants, and 1 child with ASD was taking a non‐stimulant medication for ADHD.

Edgar et al. (2023) provided details of how an ASD diagnosis was confirmed in the children with autism and how study exclusion and inclusion criteria were confirmed in the children without autism. During the course of this longitudinal study, if an autistic child later did not meet diagnostic criteria for ASD, they continued their participation in the study, as a study goal was to evaluate the relationship among neural indices and developmental trajectory. In the present study, six children with ASD did not meet diagnostic criteria for ASD at a later study time point (two children who did not meet diagnostic criteria for ASD at their second visit met criteria at a later visit). Although these six did not meet strict research criteria thresholds, they continued to display subthreshold ASD symptoms as documented by clinician judgment on the Ohio State University Autism Rating Scale (OARS; 73) and thus were still included in the ASD group.

As detailed in Table 1, the TD and ASD children did not differ in age, estimated Full Scale IQ (Wechsler Intelligence Scale for Children—Fifth Edition [WISC_V; Sattler et al. 2016]) or estimated nonverbal IQ (eNVIQ; obtained from the Fluid Reasoning Index on the WISC‐V). Groups differed on estimated verbal IQ (eVIQ; obtained from the Verbal Comprehension Index on the WISC‐V). As expected, the TD and ASD children differed on a measure of autism dimensional symptom severity, obtained by parent report on the Social Responsiveness Scale‐2 (SRS‐2) (Constantino 2012). A Fisher's Exact Test showed that the TD and ASD groups did not differ on the number of males and females (TD female = 28, ASD female = 13, *p* > 0.05). Of the combined sample, 60% were white, 16% were African American, 13% were multiracial, 8% were Asian, 1% were Pacific Islander, 1% were Hispanic, and 1% did not report their race (1 participant); 92% reported being non‐Hispanic and 8% Hispanic.

**TABLE 1:** Sample demographics.

Group	*N*	Mean	SD	*p* ^a^
Age (years)				
TD	84	11.38	2.05	0.98
ASD	63	11.39	2.03	
Estimated Full Scale Intelligence Quotient^b^				
TD	83	109.39	11.46	0.24
ASD	62	106.47	18.28	
Verbal Comprehension Index^b^				
TD	83	109.40	10.58	< 0.05
ASD	62	103.90	19.30	
Estimated Nonverbal Intelligence Quotient^c^				
TD	83	106.88	13.20	0.82
ASD	62	107.43	16.04	
Social Responsiveness Scale—2nd edition^c^				
TD	82	44.44	5.49	< 0.001
ASD	62	69.06	11.39	

^a^ Based on a *t*‐test.

^b^ Scores not available for 2 participants.

^c^ Scores not available for 3 participants.

### 2.2 MEG and MRI Data Acquisition

Edgar et al. (2023) provided details of MEG and MRI data collection. In brief, MEG data were obtained using a 275‐channel system (VSM MedTech Inc., Coquitlam, BC). In all children, after the MEG scan, sMRI provided T1‐weighted 3‐D MP‐RAGE anatomical images for source localization (3T Siemens Prisma scanner). To facilitate co‐registration of the MEG data to the child's T1 MRI, for each child three anatomical landmarks (nasion, right and left preauriculars), head position indicator locations, and scalp and face surface points were digitized using a Polhemus FastScan (Polhemus, Colchester, VT).

For the RS EC exam, over a 7‐min period, children were periodically instructed to close their eyes, receiving instructions through insert earphones to open their eyes for 10 s approximately every 2 min. If the child opened their eyes during the exam, monitored by EOG, they were reminded to close them. Excluding three 10 s eyes‐open segments, a total of 390 s of RS EC data were collected. Following the EC exam, children were administered the DR exam. For the RS DR eyes‐open exam, over a 5‐ or 7‐min period (length of DR recording was increased across the study to obtain sufficient data for subsequent functional connectivity analyses, not within the scope of this study), children were instructed to remain relaxed and keep their eyes open. For the 5‐min DR exam, the child alternated between viewing the Inscapes video (no audio) (Vanderwal et al. 2015) for 20 s and then resting with their eyes open for 30 s in total darkness sid times (the study RA was in the room with the child during the entire recording). Excluding the six 20 s Inscapes video data segments, a total of 180 s of DR eyes‐open data were collected. For the 7‐min DR exam, the child remained eyes‐open in total darkness for an additional 2 min (no Inscapes video), with a total of 300 s of DR eyes‐open data collected.

### 2.3 MEG Data Processing

MEG and sMRI data were co‐registered using BESA 6.1 and BESA MRI 3.0 (MEGIS Software GmbH, Gräfelfing, Germany). In cases where sMRI data was not evaluable due to motion or could not be collected due to scheduling conflicts (TD = 0, ASD = 2), the child's MEG data were co‐registered to an age‐matched template (Richards et al. 2016).

MEG data were processed using BESA Research 6.1. A multi‐step process was employed for removal of muscle and movement artifact. First, the child's raw EOG and MEG data were visually examined. For both the EC and DR exams, artifact correction was applied to remove heart‐beat activity, and for the DR exam, artifact correction was applied to additionally remove eye‐blink activity (Scherg and Picton 1991; Berg and Scherg 1994). For the EC exam, epochs with blinks, saccades, or other significant EOG activity were manually marked as artifacts. Then each child's MEG data in both tasks were visually inspected for muscle‐related activity, with a focus on data from sensors close to the temporalis muscles, and epochs containing muscle activity were removed. Review of the data for artifact was done blind to group status. A minimum of 80 s of artifact‐free RS data (EC and DR) was required for a child to be included in the analyses. One child had 80 s of artifact‐free data, and the remaining children had 118+ s of data.

The following pipeline was applied to the artifact‐free EC and DR data to obtain measures of brain activity in source space. To decompose the 275‐channel data into a smaller number of measures for analysis, a standard source model was applied (the BESA BRM_Brains Regions_MEG source montage), projecting each child's raw MEG sensor data into brain source space, where the waveforms are the modeled source activities. The source montage uses a set of 15 regional sources covering lateral and midline frontal cortex, central and parietal cortex, and midline fronto‐polar and occipito‐polar cortex as well as the anterior and posterior temporal lobes bilaterally. Given two orthogonal dipoles per regional source, there are two time series at each location (Hoechstetter et al. 2004). These regional sources are not intended to correspond to precise neuroanatomical structures but rather to represent neural activity at coarsely defined regions and to provide measures of brain activity with better signal separation and with a greater signal‐to‐noise ratio than would be afforded at the sensor level (Scherg and Berg 1996; Scherg and Ebersole 1993). The location of the regional sources in the model is such that there is an approximately equal distance between adjacent sources (3 cm), helping to separate signals originating from different brain regions.

To transform MEG data from the time domain to the frequency domain, a Fast Fourier Transform was applied to artifact‐free 3.41 s epochs of continuous data for each of the two orthogonally oriented time series at each regional source. Each 3.41 s epoch overlapped 50% with the next epoch, and each epoch was multiplied by a cosine squared window. This combination of overlap and windowing ensured that each time point contributed equally to the mean power spectra. When the segment of data following an artifact‐free epoch was bad, there was no overlap between epochs, and windowing was thus applied to the next available artifact‐free 3.41 s epoch. The mean power spectra for the two orthogonally oriented time series at each regional source were summed to yield the power at a given frequency at the source.

The specparam algorithm was used to parameterize the source‐space RS spectra (Donoghue, Haller, et al. 2020; Ostlund et al. 2022). In specparam, power spectra are treated as a linear combination of aperiodic activity and oscillatory peaks (individually modeled with Gaussians) that rise above the aperiodic signal. As detailed in specparam method papers (Donoghue, Haller, et al. 2020; Donoghue, Dominguez, and Voytek 2020; Ostlund et al. 2022), outputs of the specparam algorithm include the offset and exponent values of the aperiodic signal and the bandwidth, power, and center frequencies of the identified oscillatory peaks. Regression *R* ^2^ and mean absolute error measures, respectively representing the explained variance and total error of the model fit, served as goodness‐of‐fit measures used to identify potential outliers. Running the specparam model on the frequency range 2–55 Hz, review of the TD and ASD RS DR data identified the following specparam fitting parameters as consistent with those provided in previous studies (Donoghue, Haller, et al. 2020; Ostlund et al. 2022) and providing a good fit in this sample: peak width limit 1–8 Hz; max number of peaks 3; minimum peak height 0.1; proximity threshold 2; and aperiodic mode fixed. As shown in the Supporting Information, almost identical PAF, offset, and exponent values were obtained using frequency ranges of 1–55, 3–55, and 4–55 Hz.

For each child, a single PAF was determined by identifying the frequency at whichever of the parietal‐occipital sources had the highest alpha power within a 7.5–12.5 Hz range. Specifically, in each child, the PAF was determined by (1) defining alpha‐band start and end values (7.5–12.5 Hz), (2) within this frequency range identifying up to three center frequencies at nine posterior locations in specparam, and then (3) defining the PAF as the frequency at the location where the highest alpha power (power over and above the aperiodic component—the specparam peak width value) was observed. Once the brain location was identified, the PAF and periodic PAF power values were recorded. As such, for the periodic measures, each child had only a single PAF and its associated PAF power value. For aperiodic activity, to assess regional differences, offset and exponent measures were obtained from all 15 brain regions.

### 2.4 Statistical Analyses

Statistical analyses were performed using SPSS (IBM SPSS Statistics Version 29). Given a primary goal of determining the similarity of DR and EC alpha measures, analyses were run on the largest possible sample, thus including the 23 children with DR and EC measures at two or three time points. All primary analyses, however, were re‐run, removing the second or third recording for these children. The pattern of findings remained unchanged, with these analyses provided in the Supporting Information.

Although for all analyses the primary comparison was between DR and EC measures, group was included as a factor in most analyses, with the exploratory group analyses providing evidence for or against the similarity of the EC and DR group findings. Analyses were run separately for PAF, the periodic PAF power, and the aperiodic offset and exponent measures. Independent‐sample *t*‐tests examined group differences in age, estimated Full Scale IQ, eNVIQ, eVIQ, and SRS‐2. The intraclass correlation coefficient (ICC; two‐way mixed effects, absolute agreement) examined the similarity of the DR and EC alpha measures. ICC values are characterized as suggested by Cicchetti (Cicchetti 1994): < 0.70 agreement unacceptable, between 0.70 and 0.79 agreement fair; between 0.80 and 0.89 agreement good; ≥ 0.90 agreement excellent. Pearson's correlations were also computed for the DR and EC measures separately for TD and ASD, with Fisher *r*‐to‐*z* transformations assessing differences between the TD and ASD correlation coefficients. ANOVAs examined group differences in the periodic alpha frequency and power and applied Wilks' Lambda. Regressions examined associations between age and DR and EC RS periodic alpha measures, with age entered first, group second, and their interaction term last. Whereas for PAF and PAF power there was only a single value per child (obtained from the location with the highest alpha response), the aperiodic offset and exponent measures were computed separately at 15 brain regions. As such, for the aperiodic measures, a linear mixed model was run with location (15 sources) as a within‐subject factor, group a between‐subject factor, age a covariate, and offset or slope the dependent measure.

## 3 Results

### 3.1 Quality of the EC and DR Data

**FIGURE 1:**
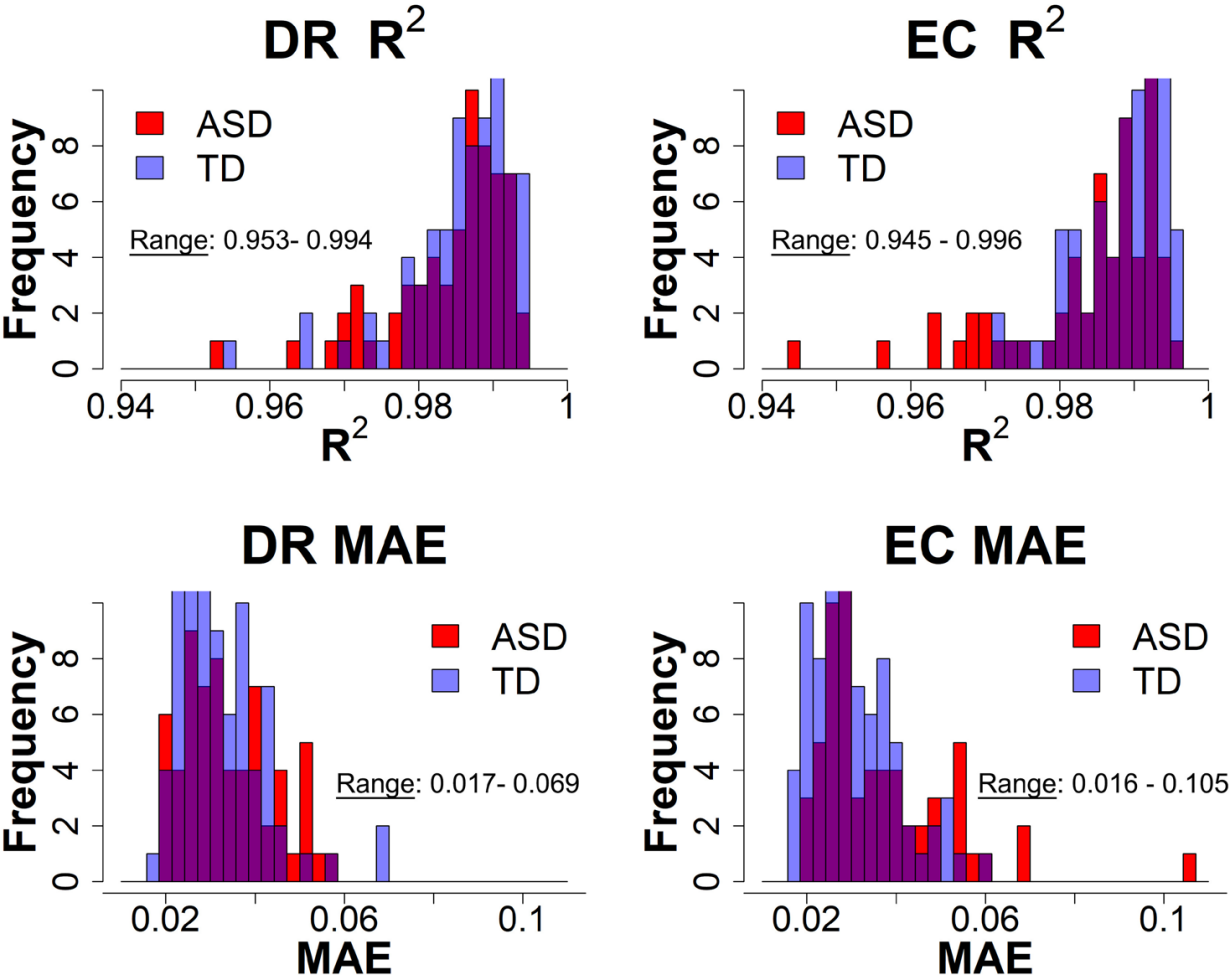
The distribution of dark room and eyes closed specparam *R* ^2^ values and mean absolute error (MAE) values for TD (blue) and ASD (red). As detailed in Ostlund et al. (2022), specparam provides two measures that describe how well the model fits the data: *R* ^2^ and MAE. MAE is computed examining the error between the original spectrum and the full model. *R* ^2^ assesses the correspondence between the original spectrum and the full model. Observed *R* ^2^ values > 0.90 and MAE values < 0.08 indicate that the model fit parameters were appropriate across the examined frequency range.

As shown in Figure 1, the specparam algorithm provided an excellent fit to the data from each child. As an example, at the PAF location most children exhibited *R* ^2^ values greater than 0.98 (range 0.945–0.996). Underfit cases (defined as *R* ^2^ < 0.95) were visually inspected and showed good reliability in correctly identifying the PAF in both conditions, so they were included in the final sample. The consistently high values indicate that the specparam power spectrum model successfully distinguished aperiodic and periodic activity. As previously noted, inspection of the DR versus EC PAF scatterplots identified four children (3 TD, 1 ASD) as multivariate outliers. As shown in the Supporting Information, almost identical *R* ^2^ values (within and across brain areas) were obtained using frequency ranges of 1–55, 3–55, and 4–55 Hz.

In assessing the amount of evaluable data, a repeated‐measures ANOVA was conducted with condition (DR, EC) as a within‐subjects measure, group as a between‐subjects measure, and percent of artifact‐free data as the dependent variable. There was no effect of condition or group (*ps* > 0.05). Collapsing across group, for the DR condition 88% of the data were evaluable (SD = 7.6, range 48%–99%), and for the EC condition 89% of the data were evaluable (SD = 9.9, range 45%–98%).

**FIGURE 2:**
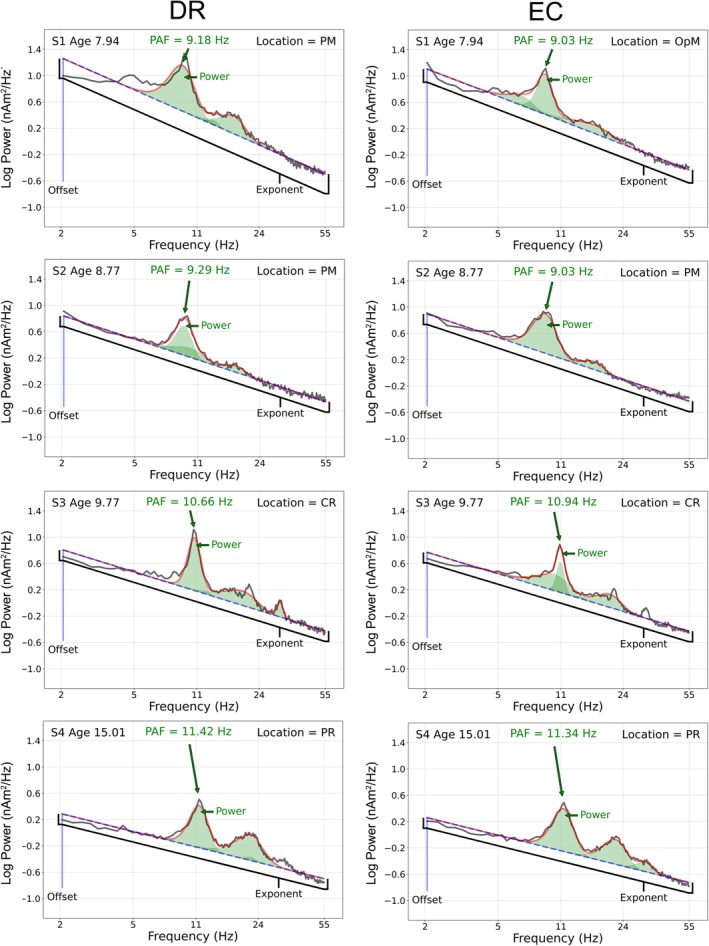
Dark room (left column) and eyes closed (right column) of the original power spectrum (black line) and specparam model (red line) in four representative children 8–15 years old. As shown in each power spectrum plot, in all children the original power spectrum (black line) is well modeled by specparam (red line). As shown in each plot, resting‐state periodic alpha activity is prominent in all children (green), observed in parietal and occipital brain regions (location for each child shown in top right of plot), and with PAF values similar between the eyes closed and dark room tasks. A higher PAF is observed in older than younger children. Aperiodic offset (y‐intercept of the specparam model fit) and exponent (slope of linear fit to the specparam model in log–log space) parameters are shown in blue.

Figure 2 shows the DR and EC specparam fits for four representative children 7–15 years old. In all children, the original power spectrum (black line) was well modeled by specparam (red line), with resting‐state periodic alpha activity prominent in all children (green), and in each child, the PAF values were similar between the eyes closed and dark room tasks.

### 3.2 Eyes‐Closed and Dark‐Room Peak Alpha Frequency and Power

Given the strong evidence that the specparam modeling was successful in separating aperiodic and periodic activity in the power spectrum, PAF was identified with the aperiodic component removed. A DR and EC PAF was observed in all children. In most children, PAF was identified in the Parietal Midline source (EC = 47.62%; DR = 42.18%) or the adjacent Occipital Midline source (EC = 29.25%, DR = 25.17%), and in nearby locations in the other children: Temporal Anterior Left (EC = 1.36%, DR = 0%), Temporal Posterior Left (EC = 0.68%, DR = 0.68%), Central Left (EC = 0.68%, DR = 2.72%), Parietal Left (EC = 5.44%, DR = 6.12%), Central Midline (EC = 3.40%, DR = 2.72%), Central Right (EC = 5.44%, DR = 9.52%), Parietal Right (EC = 4.76%, DR = 6.80%), and Temporal Posterior Right (EC = 1.36%, DR = 4.08%).

**FIGURE 3:**
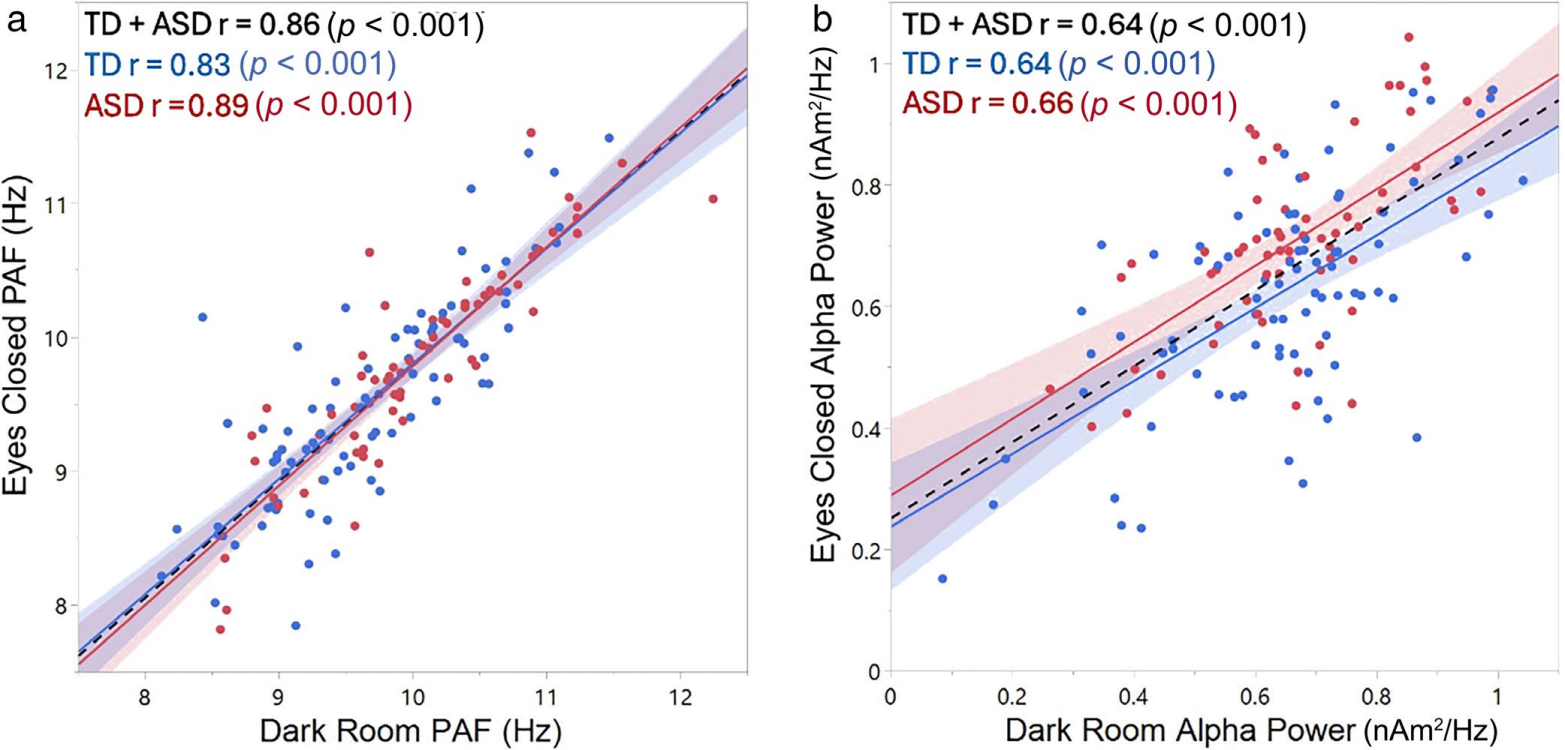
Scatterplots showing associations between dark room (DR) and eyes‐closed (EC) PAF (a) and periodic PAF power (b) for TD (blue) and ASD (red). A Fisher *r*‐to‐*z* transform showed no group differences in the DR and EC correlation coefficients.

**FIGURE 4:**
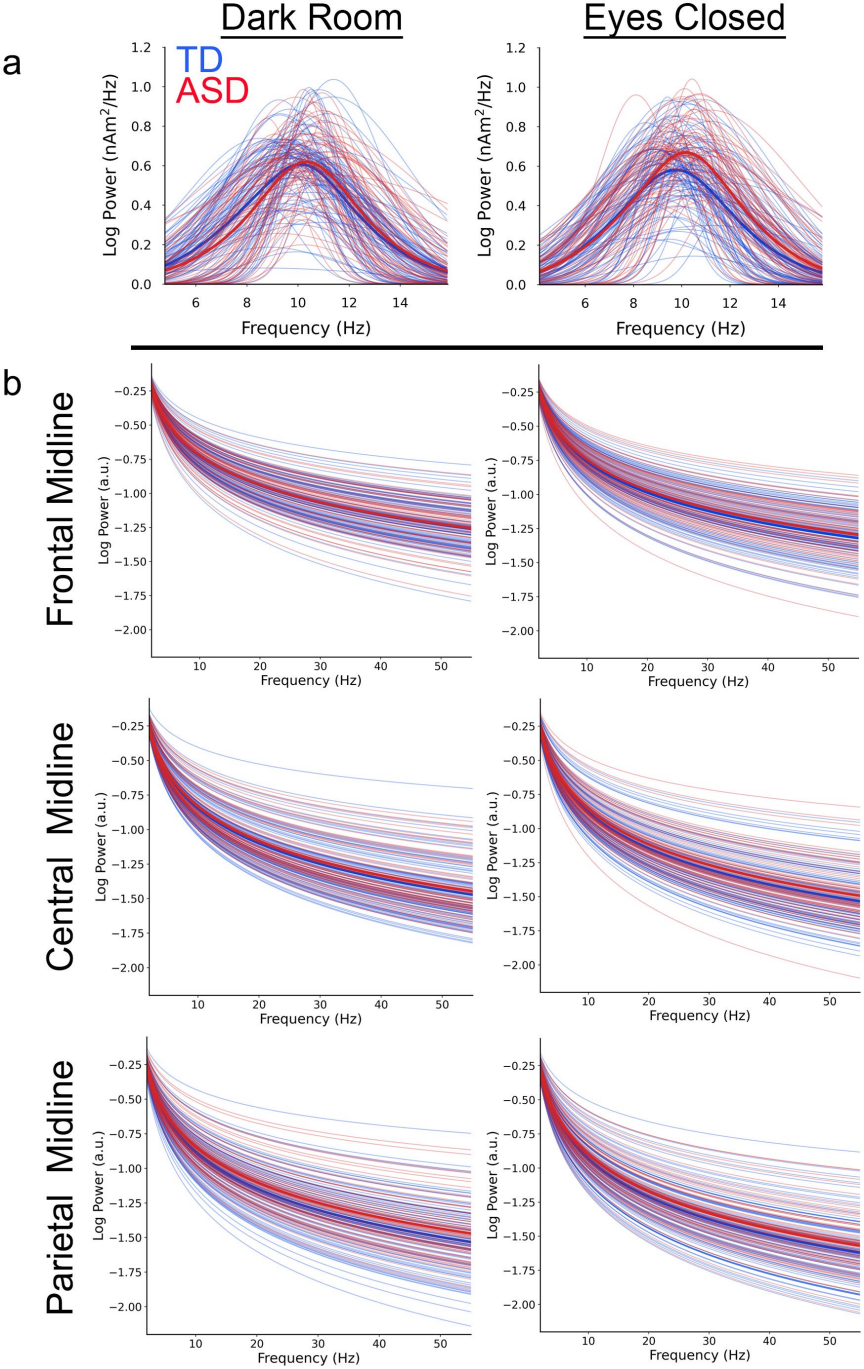
(a) Periodic DR (left) and EC (right) alpha responses for each child (TD = blue, ASD = red). Bold lines show mean value. (b) Aperiodic DR (left) and EC (right) specparam fits for each child (TD = blue, ASD = red) for frontal, central, and parietal midline locations. Bold lines show mean value.

Good reliability was observed for PAF between the DR and EC conditions: ICC Absolute Agreement (ICC) = 0.84; 95% CI 0.73–0.90, *p* < 0.001. Figure 3a shows the DR versus EC PAF scatterplot for each group with associated correlations. Correlations were high in both groups, with a Fisher *r*‐to‐*z* transformation indicating slightly stronger DR and EC associations in ASD than TD (*z* = −1.99, *p* = 0.02). Figure 4a shows periodic alpha activity for each child (TD = blue, ASD = red) for the DR (left) and EC (right) conditions.

To examine TD and ASD PAF differences, a repeated‐measures ANOVA was run with a within‐subjects factor of condition (DR, EC), a between‐subjects factor of group, and PAF as the dependent measure. PAF was consistently slightly higher in the DR condition (mean = 9.85 Hz, SD = 0.78) than in the EC condition (mean = 9.66 Hz, SD = 0.78; 95% CI = 0.12–0.26; Cohen's *d* = 0.24, *F*(1,145) = 30.42, *p* < 0.001). Collapsing across conditions, PAF was higher in ASD (mean = 9.93 Hz, SD = 0.76) than TD (mean = 9.59 Hz, SD = 0.73; 95% CI = 0.10–0.58; Cohen's *d* = 0.45, *F*(1,145) = 7.60, *p* = 0.007). The Group × Condition interaction was not significant (*p* = 0.30). Analogous results were obtained when computing the PAF from the raw power spectrum (see Supporting Information).

For PAF power, unacceptable to fair reliability between DR and EC was observed: ICC = 0.65; 95% CI 0.54–0.73, *p* < 0.001. Figure 3b shows the DR versus EC scatterplot for each group with associated correlations. A Fisher *r*‐to‐*z* transformation showed no difference between TD and ASD in DR and EC PAF power correlations (*z* = −0.20, *p* > 0.05). A simple‐effects analysis of a Group × Condition interaction (*F*(1,145) = 6.33, *p* = 0.01) showed higher PAF power in TD than in ASD in the EC condition (*p* < 0.01) and no group differences in the DR condition (*p* > 0.05), as well as condition differences in ASD (EC>DR, *p* < 0.05) but not TD (*p* > 0.05).

For the above periodic alpha analyses, and as reported in the Supporting Information, similar results were obtained when the second and third recordings from the 23 children with data from multiple time points were removed (TD = 13, ASD = 10).

### 3.3 Associations Between Age and Eyes‐Closed and Dark‐Room Peak Alpha Frequency and Power

#### 3.3.1 PAF

**FIGURE 5:**
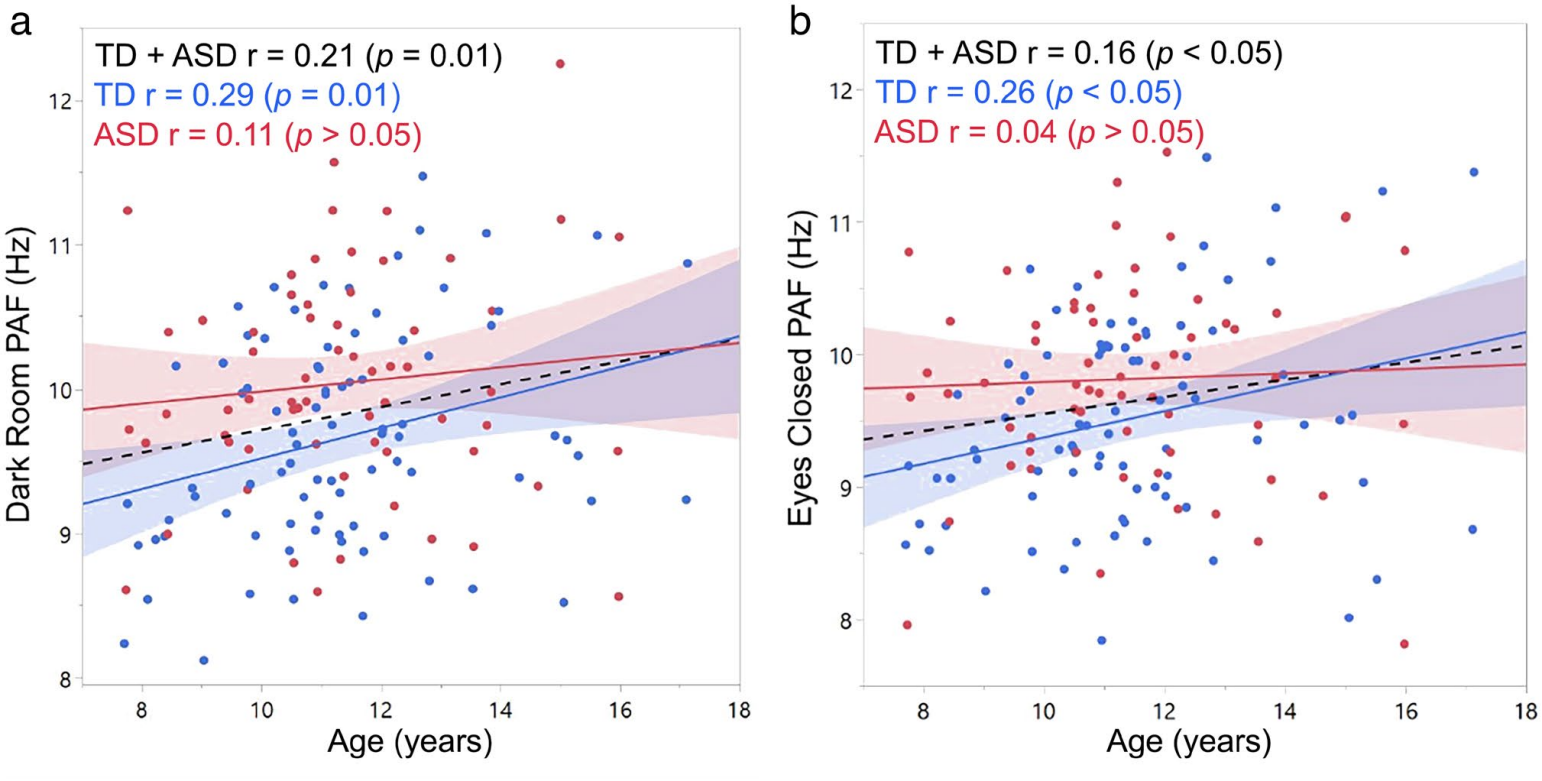
Scatterplots showing associations between age and PAF for TD (blue) and ASD (red) for the dark room (DR, a) and eyes closed (EC, b) conditions. For both DR and EC, a main effect indicated a higher PAF in older than younger children. *R*‐values and their associated *p*‐values are also shown for the TD and ASD children.

The Figure 5 scatterplots show associations between age (*x* axis) and PAF (*y* axis) for DR (left panel) and EC (right panel). For both DR and EC conditions, main effects of age (*p*s < 0.05) showed positive association between age and PAF for the full sample. For both conditions, the group difference in PAF (ASD > TD) remained (*ps* < 0.05) after removing variance in PAF associated with age. The interaction terms were not significant.

#### 3.3.2 Periodic Power

For the DR condition, no main effects or interactions were observed. For the EC condition, simple effect analysis of a Group × Age interaction, *F*(1,143) = 9.50, *p* < 0.01, showed a negative association between age and EC PAF power for TD (*r* = −0.33, *p* < 0.01) versus a nonsignificant positive association for ASD (*r* = 0.17, *p* > 0.05).

As reported in the Supporting Information, analogous periodic alpha frequency and power results were obtained when the second recording from the 23 children with data from multiple time points was removed.

### 3.4 Correlations Between EC and DR Aperiodic Measures

As shown in Table 2, across the 15 brain regions all DR and EC ICC offset and exponent values were significant (all *ps* < 0.001), with ICC values in the full sample in the fair to good range: mean ICC = 0.77, median ICC = 0.80. Although Fisher *r*‐to‐*z* transformation showed some differences between TD and ASD in the DR and EC offset and exponent correlation coefficients, no clear pattern was evident, with the correlation values sometimes larger in TD than ASD and sometimes larger in ASD than TD. Figure 4b shows aperiodic activity for each child (TD = blue, ASD = red) for the DR (left) and EC (right) conditions.

**TABLE 2:** Correlation between Dark room and eyes closed offset (top rows) and exponent (bottom rows) for each region.

Region	Offset total sample^a^	Offset TD^a^	Offset ASD^a^
Temporal anterior left	0.81	0.85	0.73
Temporal parietal left	0.80	0.78	0.83
Frontal left	0.86	0.86	0.86
Central left	0.84	0.83	0.85
Parietal left	0.85	0.83	0.87
Fronto‐polar midline	0.76	0.78	0.73
Frontal midline	0.80	0.82	0.79
Central midline	0.81	0.79	0.83
Parietal midline	0.86	0.83	0.89
Occipito‐polar midline	0.72	0.67	0.77
Frontal right	0.81	0.83	0.78
Central right	0.90	0.90	0.89
Parietal right	0.85	0.83	0.87
Temporal anterior right	0.80	0.81	0.79
Temporal parietal left	0.86	0.86	0.85

Region	Exponent total sample^a^	Exponent TD^a^	Exponent ASD^a^
Temporal anterior left	0.69	0.78	0.49
Temporal parietal left	0.61	0.63	0.58
Frontal left	0.76	0.77	0.74
Central left	0.76	0.76	0.76
Parietal left	0.76	0.79	0.71
Fronto‐polar midline	0.75	0.77	0.73
Frontal midline	0.72	0.72	0.72
Central midline	0.82	0.83	0.81
Parietal midline	0.81	0.85	0.75
Occipito‐polar midline	0.62	0.69	0.82
Frontal right	0.61	0.66	0.53
Central right	0.77	0.81	0.74
Parietal right	0.80	0.86	0.66
Temporal anterior right	0.56	0.64	0.46
Temporal parietal left	0.61	0.66	0.51

^a^ All significant at *p* < 0.001.

### 3.5 Associations Between Age and Aperiodic Measures

**FIGURE 6:**
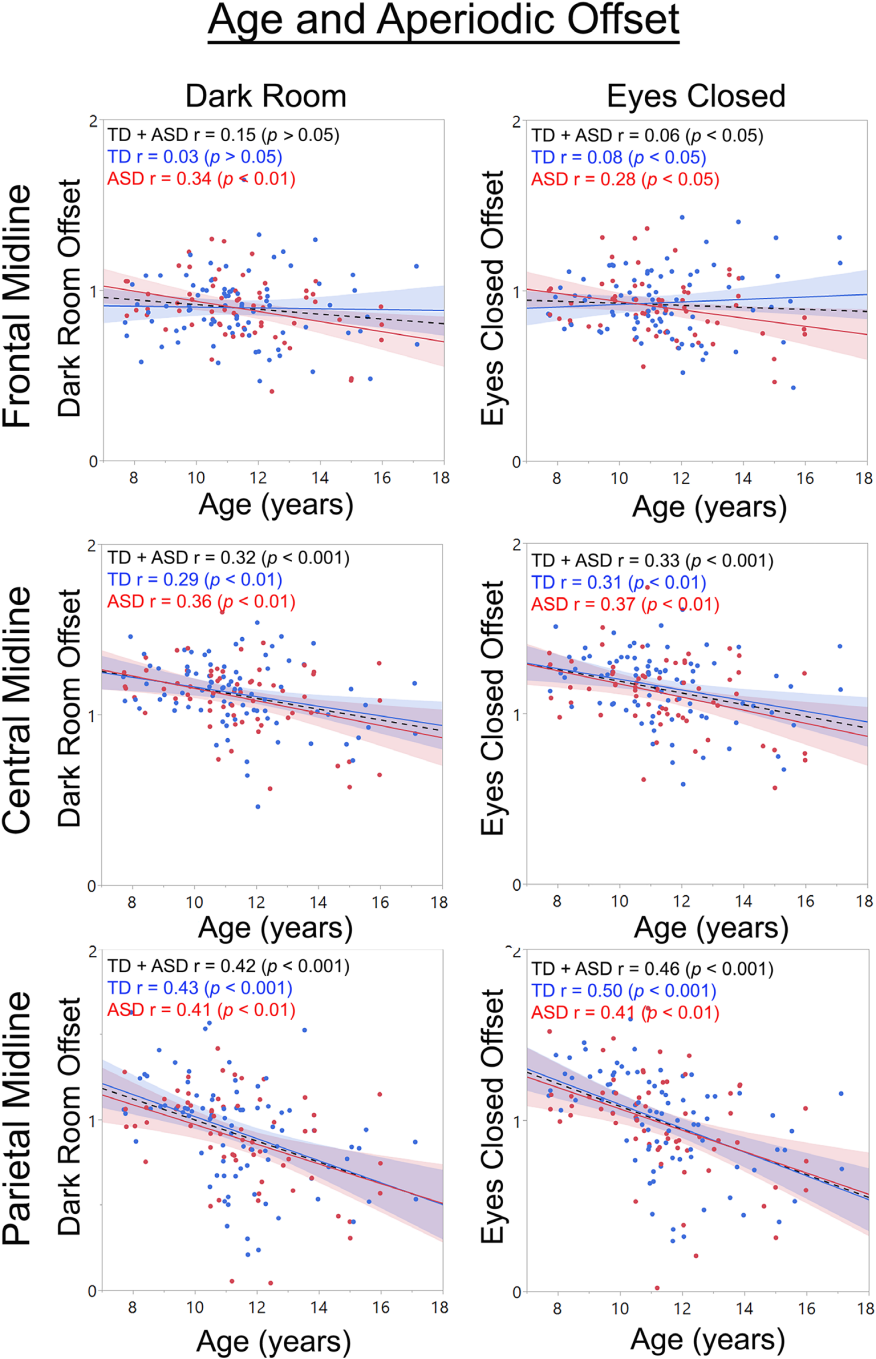
Scatterplots showing age and offset associations for DR and EC tasks for frontal midline, central midline, and parietal midline locations. Age × Region interactions indicated that offset values and age and offset associations differed among the 15 regions; 3 midline regions are shown here.

**FIGURE 7:**
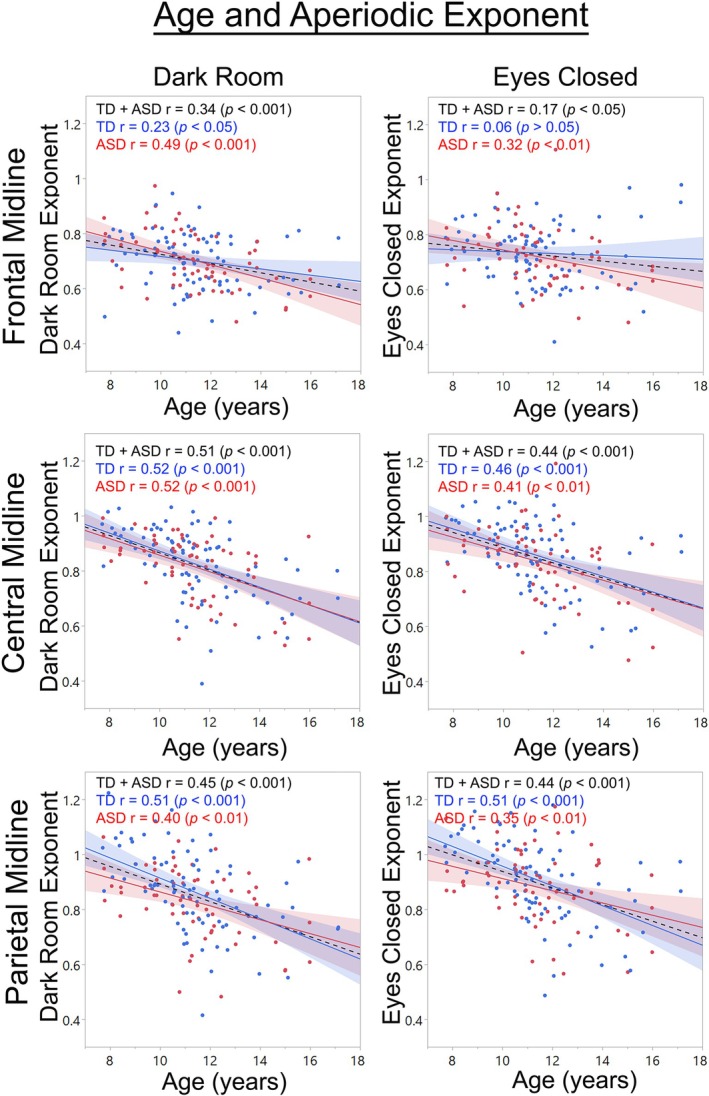
Scatterplots showing age and exponent associations for DR and EC tasks for frontal midline, central midline, and parietal midline locations. Age × Region interactions indicated that offset values and age and offset associations differed among the 15 regions; 3 midline regions are shown here.

Figures 6 and 7 show associations between age and DR offset, EC offset, DR exponent, and EC exponent aperiodic parameters at 3 midline sources. For the DR and EC offset measures, main effects of group (*ps* < 0.01) showed higher offset values in TD than ASD (DR TD mean = 1.033, SD = 0.270, ASD mean = 1.001, SD = 0.270, Cohen's *d* = 0.12; EC TD mean = 1.033, SD = 0.270, ASD mean = 1.001, SD = 0.270, Cohen's *d* = 0.12). Group × Age interactions (*ps* < 0.01) showed stronger offset and age associations in ASD (DR *r* = 0.34, EC *r* = 0.34, *ps* < 0.001) than TD (DR *r* = 0.22, EC *r* = 0.22, *ps* < 0.001). Main effects of region were qualified by Region × Age interactions (*ps* < 0.001). As examples, and as shown in Figure 6, age and offset associations were stronger in posterior than midline than frontal regions.

For the DR and EC exponent measures, main effects of group and region were qualified by Group × Region × Age interactions (*ps* < 0.001). As examples, and as shown in Figure 7, age and exponent associations were generally stronger in posterior than midline and frontal regions and differed between TD and ASD (e.g., EC frontal midline), and with regional differences in the exponent values apparent in the scatterplots.

As reported in the Supporting Information, analogous aperiodic results were obtained when the recordings from the 23 children with data from multiple time points were removed, with the exception that for the aperiodic offset no group differences were observed in either condition.

## 4 Discussion

In a larger sample and a wider age range than Edgar et al. (2023), good correspondence between RS DR and EC parietal‐occipital periodic PAF measure in TD and ASD children was again observed. Different from Edgar et al., the present study examined the reliability of RS aperiodic offset and exponent values at 15 brain regions. Fair to good correspondence between the RS DR and EC offset and exponent values in TD and ASD was observed at all regions. The aperiodic offset and exponent values differed across the 15 brain regions, as did associations between age and the aperiodic measures (e.g., Figures 6 and 7). Present findings demonstrate that the DR exam is a viable way to obtain RS periodic and aperiodic measures. Regional differences in aperiodic values as well as regional differences in associations with age demonstrate the need to assess aperiodic activity in brain versus sensor space, which obscures regional differences.

### 4.1 Resting‐State Periodic Alpha Findings

Good reliability was observed for the DR and EC PAF (full sample ICC = 0.84). The observation of higher PAF in ASD than TD is in line with our previous studies, observed in two different cohorts (Edgar et al. 2015, 2019) and a slightly overlapping cohort (TD = 11% and ASD = 7%; (Shen et al. 2023)). For DR and EC PAF power, ICC analyses showed moderately unacceptable to fair reliability (full sample ICC = 0.65).

The DR PAF group findings comparisons mirrored the EC PAF group findings and thus in addition to the high ICCs provide additional support for use of the DR task for assessing resting‐state neural activity. In particular, in the present study, the age and PAF regressions replicated studies showing a higher PAF in older than younger children (e.g., (Miskovic et al. 2015; Somsen et al. 1997; Chiang et al. 2011)). Figure 5 age and PAF scatterplots show the similarity of the age and DR PAF and age and EC PAF associations, as well as larger correlation values in TD than ASD. Although the Age × Group interaction was not significant, the evident TD and ASD differentiation is similar to those reported in larger sample studies, with age‐related changes in resting‐state PAF observed in TD but not ASD children (Edgar et al. 2015, 2019; Dickinson et al. 2018). For example, in a sample of 121 TD and 183 ASD male children 6‐ to 17‐years‐old (none in the sample reported in this study), Edgar et al. (2019) found a significant association between age and PAF in TD (*r* = 0.56) but not ASD (*r* = 0.10). The Edgar et al. TD and ASD *r* values are similar to those in the present study for age and PAF in the DR (TD *r* = 0.29; ASD *r* = 0.11) and EC conditions (TD *r* = 0.26; ASD *r* = 0.04). Such parallels provide additional evidence in support of the similarities in the DR and EC alpha measures.

### 4.2 Resting‐State Aperiodic Findings

Fair to good reliability was observed for the DR and EC aperiodic offset and exponent values at the 15 brain regions (see Table 2). The DR and EC group and age findings were generally similar, providing further support of the DR task.

Present aperiodic reliability was higher than that of Levin et al. (2020), who reported reliability values for aperiodic measures in children with and without autism that most scientists would deem unacceptable for use as a biomarker (their ICCs ranged from 0.28 to 0.70; see their Table 4). The present study compared two tasks in a single session vs. Levin et al. comparing RS data collected a median of 6 days apart. Also, the present study assessed eyes‐closed/dark‐room RS activity versus eyes‐open activity in Levin and evaluated RS activity in brain space (here) versus sensor space (Levin). Studies examining aperiodic activity in brain space across time (including recordings separated by days or weeks) need to better assess the reliability of RS aperiodic activity.

For parameterized RS data, also of note is the benefit of neurophysiological measures that are reference‐free. Measurement of the exponent (slope) of the RS power spectrum is hypothesized to provide a non‐invasive measure of the neural‐circuit excitatory:inhibitory (E:I) balance (Gao et al. 2017). Valid determination of this value using EEG sensor measures is complicated by the fact that different referencing strategies, such as bipolar versus common average, produce very different exponent estimates (Shirhatti et al. 2016). The RS power spectrum exponent and offset parameters can be more directly measured via source‐space analyses.

Pediatric studies examining RS aperiodic activity have shown that from childhood to late adolescence the RS 1/f power‐spectrum slope flattens as a function of age, eventually stabilizing at an adult level (Schaworonkow and Voytek 2021; Karalunas et al. 2022; Tran et al. 2020; Waschke et al. 2017; Voytek et al. 2015). Present findings (see Figures 6 and 7) replicate and extend this literature, here showing that the offset and exponent values differ by brain region and also showing that the offset and exponent age‐related associations differ among the 15 brain regions. The finding of higher offset values in TD than ASD, and for the exponent measure, a 3‐way Group × Region × Age interaction are taken as preliminary; these findings indicate that the group differences in aperiodic activity are perhaps specific to a given brain area and/or age.

As noted in the Introduction, in an MEG study Vandewouw et al. (2024) showed offset and exponent regional differences in children and adults 1–38 years old. More recently, Green et al. (2025) found in children 0–5 years old regional differences in the offset and exponent values. Present and recent findings raise concerns regarding assessing aperiodic EEG or MEG sensor activity, with sensor measures likely reflecting activity from multiple brain regions (e.g., for discussions of the problems associated with this approach, see Hoechstetter et al. 2004; Scherg and Berg 1996; Scherg and Picton 1991; Edgar et al. 2017; Edgar et al. 2003). As an example, Schaworonkow and Nikulin (2022) illustrated that, due to volume conduction, RS alpha activity from occipital, sensorimotor, and superior temporal gyrus neural sources is mixed at the level of the EEG/MEG sensor, with assessment of frontal EEG sensors showing that the contribution of occipital and sensorimotor neural generators can be as high as 75%, making inferences about the activation of frontal cortex activity based on frontal electrode activity problematic.

Present regional differences in aperiodic offset and exponent measures as well as in associations with age are hypothesized to be due, in part, to regional differences in brain maturation. For example, gray‐matter volume increases rapidly in young childhood, peaking between 5 and 6 years of age before decreasing nonlinearly (Bethlehem et al. 2022). In young infants, gray‐matter growth rates are regionally specific, with primary sensory, occipital, and parietal regions maturing earlier than prefrontal regions (e.g., Gilmore et al. 2007; Gogtay et al. 2004). White‐matter volume also increases from infancy to young adulthood before peaking between 28 and 29 years old (Bethlehem et al. 2022), with white‐matter myelination occurring first in primary sensory cortices and myelination of prefrontal and association cortices occurring later in infancy and continuing through late childhood (Gilmore et al. 2007; Deoni et al. 2015; Ouyang et al. 2019). Studies assessing associations between local RS aperiodic activity and local brain structure are of interest.

### 4.3 Limitations and Future Directions

A study limitation is that the present sample of children with autism included many with low support needs (although per Table 1 they had somewhat lower mean eNVIQ scores than the TD group, as is common). Studies obtaining DR data in children with autism with greater support needs are of interest to determine whether present group differences are observed across the autism spectrum. Our experience to date indicates that the DR task is tolerated across the lifespan and can be used in children with greater support needs. We have used the DR task to describe the maturation of RS neural activity in 102 infants (Green et al. 2025), and we are collecting DR data in children born very to extremely premature who are now 3–5 years old (to date 21 premature children and 25 control children). In addition to being well tolerated, we have found that the DR task may be preferred in adolescents, who often stay up late and arrive to their brain imaging appointment sleep‐deprived, sometimes falling asleep during the eyes‐closed exam (thus showing no resting‐state alpha rhythm as well as significantly altering aperiodic background activity), but much more likely to stay awake during the eyes‐open DR task.

Another study limitation is that given the study design the EC task was always administered before the DR task. Future studies should counterbalance the conditions.

## 5 Conclusions

Present results replicate and extend previous studies showing that a DR eyes‐open exam provides RS periodic and aperiodic measures similar to those obtained in an RS EC exam. Given regional differences in the aperiodic offset and exponent measures, measuring aperiodic activity in brain space rather than sensor space is needed to more precisely assess RS activity.

## Author Contributions

**Marybeth McNamee:** writing – original draft, writing – review and editing, formal analysis. **Heather L. Green:** investigation, writing – original draft, writing – review and editing, formal analysis, project administration, supervision. **Guannan Shen:** formal analysis. **Marissa DiPiero:** investigation, writing – original draft, writing – review and editing. **Drayton L. Murray:** investigation, writing – original draft, methodology, writing – review and editing, formal analysis. **Mia Pearce:** writing – original draft, formal analysis, investigation, visualization. **Alice Onyango‐Opiyo:** writing – original draft, formal analysis. **Song Liu:** formal analysis, writing – review and editing, software. **Lisa Blaskey:** investigation, writing – original draft, methodology, writing – review and editing, project administration, supervision. **Emily S. Kuschner:** writing – review and editing, project administration, supervision. **Mina Kim:** writing – review and editing, supervision. **Rose E. Franzen:** investigation, writing – review and editing, formal analysis, visualization. **Gregory A. Miller:** writing – original draft, writing – review and editing, formal analysis, supervision. **Yuhan Chen:** writing – original draft, writing – review and editing, formal analysis, supervision, data curation, funding acquisition. **J. Christopher Edgar:** conceptualization, investigation, funding acquisition, writing – original draft, methodology, validation, writing – review and editing, formal analysis, project administration, supervision, resources.

## Funding

This study was supported in part by NIH grant R01MH107506 (J.C.E.) (R21MH098204, R21NS090192), NICHD grant R01HD093776 (J.C.E.), NICHD grant R01HD119165 (YC), TALK Supplement Grant GRT‐00003651 (Y.C.), and NICHD grant K08HD114880 (H.L.G.).

## Conflicts of Interest

The authors declare no conflicts of interest.

## Supporting information


**Data S1:** psyp70261‐sup‐0001‐Supinfo.docx.

## Data Availability

The datasets generated and/or analyzed will be made available upon completion of this study and reasonable request.

## Endnotes

^1^
Individuals on the autism spectrum, their parents, and professionals in the field differ regarding the use of person‐first (e.g., children with ASD) or identity first (e.g., autistic child) language (Sauseng et al. 2008). With respect for divided opinions, both approaches to terminology are used here.
