# Supplementary material for: Confirmation of a Useful Dark‐Room Resting‐State Procedure: Periodic and Aperiodic MEG Activity in Children
Source: Psychophysiology. 2026 Feb 22;63(2):e70261. doi: 10.1111/psyp.70261 (PMC12926522; doi:10.1111/psyp.70261)
Supplement: Supplementary file 1 — Data S1: psyp70261‐sup‐0001‐Supinfo.docx. [file PSYP-63-e70261-s001.docx]

**Analyses removing participants with 2^nd^ or 3^rd^ resting-state recording**

Primary analyses we rerun removing for 20 subjects their 2^nd^ dataset and for 3 subjects their 2^nd^ and 3^rd^ datasets. A Fisher’s Exact Test showed that the TD (N = 69) and ASD (N = 53) groups did not differ on the number of males and females (TD female = 25, ASD female = 12, *p* > 0.05).

**3.2** **Eyes-closed and Dark-room Peak Alpha Frequency and Power**

Good reliability was observed for PAF between the DR and EC conditions: ICC = 0.83; 95% CI 0.69 to 0.90, *p* < 0.001.

To examine TD and ASD PAF differences, a repeated-measures ANOVA was run with a within-subjects factor of condition (DR, EC), a between-subjects factor of group, and PAF as the dependent measure. PAF was consistently slightly higher in the DR condition (average = 9.84 Hz, SD = 0.79) than in the EC condition (average = 9.63 Hz, SD = 0.77; 95% CI = 0.14 to 0.29; Cohen’s *d* = 0.27, F(1,120) = 33.40, *p* < 0.001). Collapsing across conditions, PAF was higher in ASD (average = 9.93 Hz, SD = 0.80.73) than TD (average = 9.54 Hz, SD = 0.73; 95% 0.10 to 0.58; Cohen’s *d* = 0.51, F(1,120) = 8.51, *p* = 0.004). The Group x Condition interaction was not significant (*p* = 0.45).

For PAF power, unacceptable to fair reliability between DR and EC was observed: ICC = 0.67; 95% CI 0.56 to 0.76, *p* < 0.001). A repeated-measures ANOVA examined group differences in PAF power differences. A simple-effects analyses of a Group x Condition interaction (F(1,120) = 4.16, *p* < 0.05), showed higher PAF power in TD than ASD in the EC condition (*p* < 0.05) and no group differences in the DR condition (*p* > 0.05), as well as trending condition differences in ASD (EC > DR, *p* = 0.09) but not TD (*p* > 0.05).

**Associations between Age and Eyes-closed and Dark-room Peak Alpha Frequency and Power**

**PAF:** For both the DR and EC condition, trending main effects of age (DR *p* = 0.06; EC *p* = 0.16) showed a positive association between age and PAF for the full sample. For both conditions, main effects of group (*ps* < 0.05) showed that the group difference in PAF (ASD > TD) remained after removing variance in PAF associated with age. The interaction terms were not significant.

**Periodic power:** For the DR condition, no main effects or interactions were observed. For the EC condition, simple effect analysis of a Group x Age interaction, F(1,118) = 6.06, *p* < 0.05, showed a negative association between age and EC PAF power for TD (r = 0.30, *p* = 0.01) versus a non-significant positive association for ASD (r = 0.11, *p* > 0.05.)

**Correlations between EC and DR Aperiodic Measures**

Across the 15 brain regions the DR and EC ICC Absolute Agreement offset and exponent values were all significant (all *ps* < 0.001), with the ICC values ranging from 0.59 to 0.83.

**Associations between Age and Aperiodic Measures**

For the DR and EC offset measures, main effects of region and age were qualified by a region x age interaction (almost all pairwise *p*-values <0.001). No other effects were significant. Offset and exponent decreased as a function of age, with regional differences in these associations. The main effect of group and group interaction terms were not significant.

For the DR and EC exponent measures, main effects of group and region were qualified by group x region x age interactions (*ps* < 0.001).

**PAF computed from raw power spectrum: assessing condition and group differences**

To examine TD and ASD PAF differences (with PAF computed from their raw power spectra), a repeated-measures ANOVA was run with a within-subjects factor of condition (DR, EC), a between-subjects factor of group, and PAF as the dependent measure. PAF was slightly but consistently higher in the DR condition (mean = 9.89 Hz, SD = 0.88) than in the EC condition (mean = 9.70 Hz, SD = 0.84; 95% CI = 0.11 to 0.30; Cohen’s *d* = 0.22, *p* < 0.001). Collapsing across conditions, PAF was higher in ASD (mean = 9.94 Hz, SD = 0.89) than TD (mean = 9.65 Hz, SD = 0.81; 95% 0.02 to 0.57; Cohen’s *d* = 0.34, *p* = 0.04). The Group x Condition interaction was not significant (*p* = 0.11).

**Similarity of specparam values across frequency range**

The similarity of the PAF, offset and exponent measures was examined running specparam for several different frequency ranges. The peak width limit, max number of peaks, minimum peak height, and proximity threshold were left unchanged, as these settings likely not only work well to identify parietal-occipital alpha peaks, but to identify periodic peaks in other brain regions. The ‘max number of peaks’ setting of 3 was expected to be liberal, allowing the possibility of finding multiple peaks in all examined brain regions. The ‘peak width limit’ of 1 to 8 Hz was also considered liberal, allowing the possibility of broad periodic peaks in all examined brain regions. In these tests, the aperiodic mode setting was always ‘fixed’ (i.e., without knee). In a paper we hope to submit later, we will present knee and no-knee comparisons.


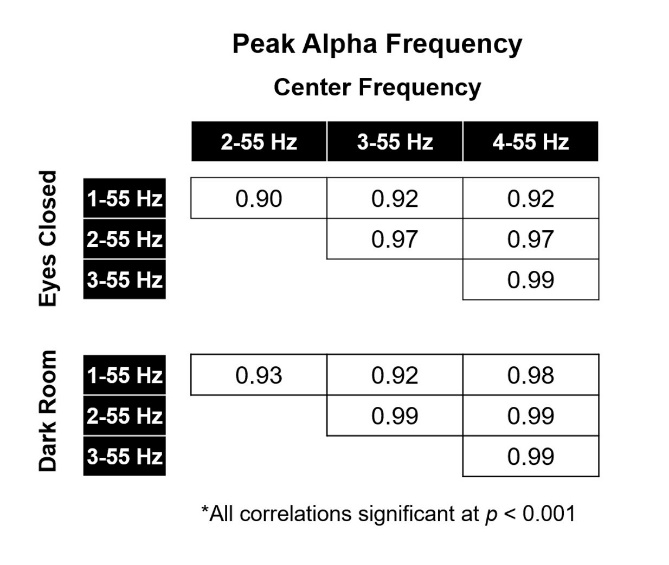


**Specparam findings using different frequencies:** Tests showed that the PAF, offset, and exponent values were very similar across the 4 examined frequency ranges: 1-55, 2-55, 3-55, and 4-55 Hz. For the PAF (see left), pairwise correlations between the PAF center frequency measured at each frequency range showed strong associations for the EC task (range of r values = .90–.99; *ps* < 0.001) and the DR task (range of r values = .92–.99; *ps* < 0.001), thus showing that the same PAF was identified across the four broader frequency ranges.


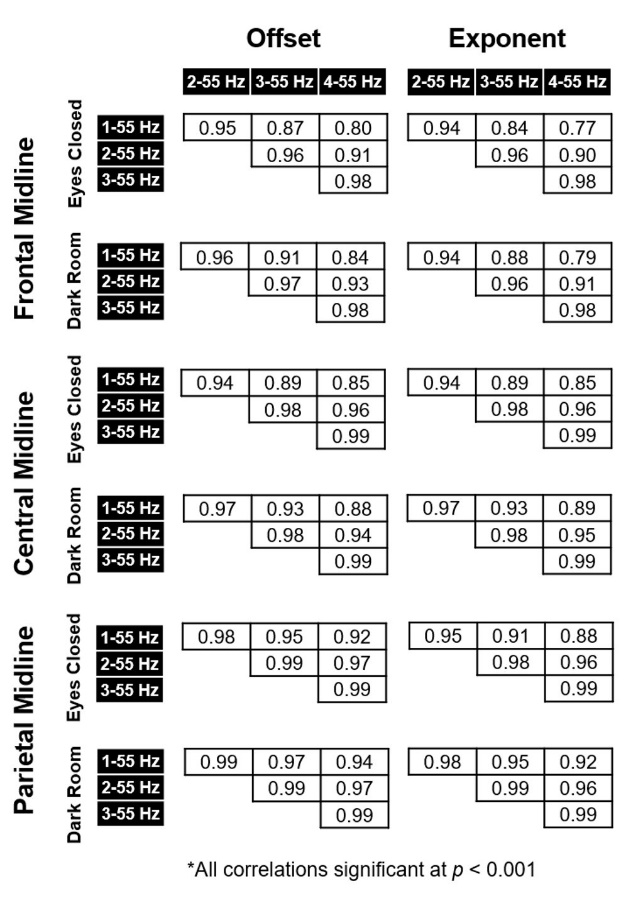
For the aperiodic measures, similarities across frequency ranges were examined at 3 midline locations: frontal (FM), central (CM), and parietal midlines (PM). High correlations were observed for the aperiodic offset at the 3 midline locations: FM (EC = .80–.98; DR = .84–.98), CM (EC = .85–.99; DR = .88–.99), and PM (EC = .92–.99; DR = .94–.99). Analogous results were observed for the aperiodic exponent: FM (EC = .77–.98; DR = .79–.98), CM (EC = .85–.99; DR = .89–.99), and PM (EC = .88–.99; DR = .92–.99). All correlations were significant at *p* < 0.001. These findings indicated that the same offset and exponent value were identified across the four frequency ranges.


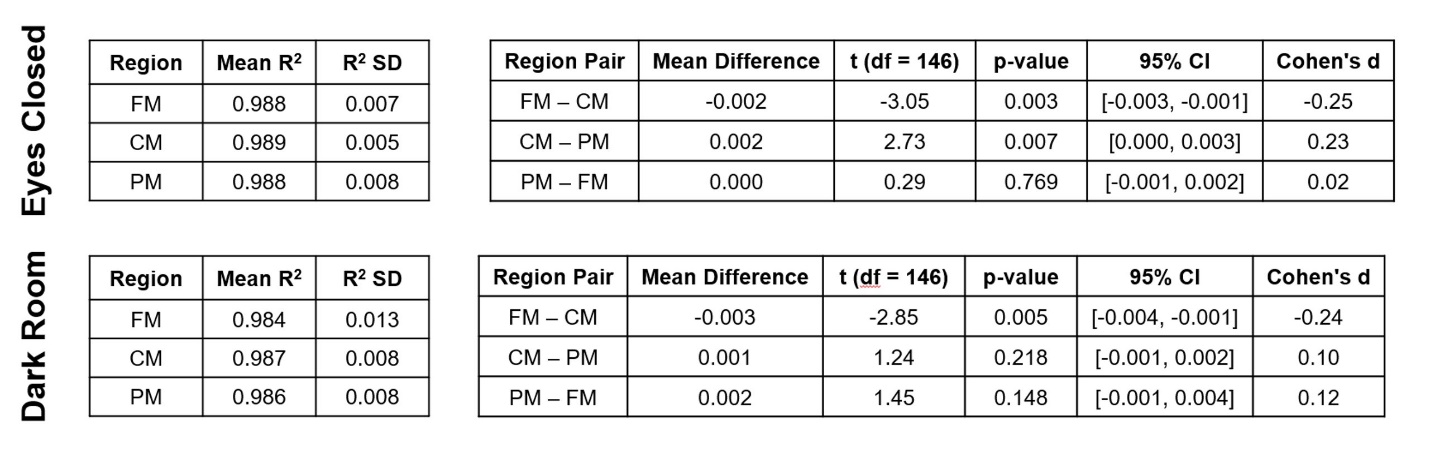
**Specparam model fits:** The quality of the model fits, here examined using the specparam R^2^ value, was examined across the 3 midline brain regions. Model fits were consistently very high within and between all 3 brain regions. Although some of the between-region comparisons showed significant R^2^ differences, the Cohen’s d values show that these differences were very small. Thus, although statistically detectable, regional differences in model fit were negligible.

To our mind, given such small R^2^ differences across brain areas, and given the very large R^2^ values, it will often be difficult to identify an ‘optimal’ model, with slight tweaks to the specparam settings perhaps providing statistically significant different R^2^ value at the same or different locations, but with the importance of these differences difficult to determine.
